# Variations in the Prevalence of Risk Factors for Coronary Artery Disease in Rio Grande do Sul-Brazil: A Comparative Analysis between 2002 and 2014

**DOI:** 10.5935/abc.20150127

**Published:** 2015-12

**Authors:** Iseu Gus, Rodrigo Antonini Ribeiro, Sérgio Kato, Juliano Bastos, Claudio Medina, Claudio Zazlavsky, Vera Lucia Portal, Rita Timmers, Melissa Medeiros Markoski, Carlos Antônio Mascia Gottschall

**Affiliations:** 1 Instituto de Cardiologia do Rio Grande do Sul - Fundação Universitária de Cardiologia, Porto Alegre, RS - Brazil; 2 Universidade Federal do Rio Grande do Sul - UFRGS, Porto Alegre, RS - Brazil; 3 Secretaria Estadual da Saúde do Rio Grande do Sul, Porto Alegre, RS - Brazil; 4 Universidade Federal de Ciências da Saúde de Porto Alegre - UFCSPA, Porto Alegre, RS - Brazil; 5 Pontifícia Universidade Católica do Rio Grande do Sul - PUCRS, Porto Alegre, RS - Brazil

**Keywords:** Risk Factors, Prevalence, Coronary Artery Disease / epidemiology, Comparative Study

## Abstract

**Background:**

Due to the importance of coronary artery disease (CAD), continuous investigation of the risk factors (RFs) is needed.

**Objective:**

To evaluate the prevalence of RFs for CAD in cities in Rio Grande do Sul State, and compare it with that reported in a similar study conducted in the same cities in 2002.

**Methods:**

Cross-sectional study on 1,056 healthy adults, investigating the prevalence and absolute and relative frequencies of the following RFs for CAD: obesity, systemic arterial hypertension (SAH), dyslipidemias, smoking, sedentary lifestyle, diabetes mellitus, and family history, as well as age and sex. Data was collected in 19 cities, host of the Offices of the Regional Coordinators of Health, as in the 2002 study.

**Results:**

Twenty-six percent of the sample consisted of older adults and 57% were women. The prevalence of sedentary lifestyle was 44%, history family 50%, smoking 23%, overweight/obesity 68%, dyslipidemia (high cholesterol levels) 43%, SAH 40%, and diabetes 11%. When compared to the 2002 study, the prevalence of active smoking and sedentary behavior decreased, whereas the prevalence of hypertension, dyslipidemia and obesity increased. Obesity is the most prevalent RF in women, and SAH the most prevalent in men.

**Conclusions:**

The prevalence of RFs for CAD in Rio Grande do Sul State remains high. Hypertension, obesity and dyslipidemia are still prevalent and require major prevention programs. Smoking and physical inactivity have decreased in the state, suggesting the efficacy of related campaigns.

## Introduction

In Brazil, coronary artery disease (CAD) is a class of cardiovascular disease (CVD) with high hospitalization rates and high costs^1-4^. CAD remains as one of the major causes of mortality in developing countries, reaching an incidence and prevalence of epidemic proportions in several parts of the world^4-15^. In light of such situation, continuous study of the known risk factors (RFs) for CAD is needed. The main RFs are systemic arterial hypertension (SAH), diabetes mellitus, sedentary lifestyle, smoking, dyslipidemia, obesity and genetic factors (family history). These factors may be managed (to a better or worse condition) by several interventions, including pharmacotherapy, diet, physical activity and behavior change.

The population of the south of Brazil stands out among other regions of the country for their high life expectancy^8,16^. On the other hand, this leads to a rapid emergence of chronic non-communicable diseases^16^, commonly associated with unhealthy habits such as high-fat diet, and low level or no physical activity. Therefore, this population is highly likely to develop CVDs including CAD.

In a study conducted in 2002, 19 cities hosting the Offices of the Regional Coordinators of Health were screened for the prevalence of RFs in Rio Grande do Sul State^6^. The results showed that sedentary lifestyle, history family of CAD and obesity were present in more than 50% of the state’s population. Aiming to investigate the current prevalence of RFs in Rio Grande do Sul State and to compare it with data from 2012, the same 19 cities were screened in 2014. Although different subjects were included in the 2014 study, all of them were residents of the same locations. Here, we analyze and discuss the data obtained in 2014; this data may contribute to the planning of new primary prevention programs in Rio Grande do Sul State in the future.

## Methods

### Study design and variables of interest

This was a cross-sectional study with analysis of absolute and relative frequencies of FRs. The reference values for the RFs (SAH, diabetes, sedentary lifestyle, smoking and obesity) were established according to WHO recommendations and previous reports^17,18^. Body mass index (BMI) was used for the diagnosis of obesity; dyslipidemia was defined as total cholesterol above 200 mg/dL; a blood pressure greater than 140/90 mmHg was considered for SAH, and glucose levels greater than 120 mg/dL for diabetes; for smoking, only currently (active) smokers were considered; sedentary lifestyle was defined as exercising less than twice a week, and only first degree relatives were considered for analysis of family (genetic) background.

In addition, the study investigated the level of patients’ knowledge about their condition concerning the presence of diabetes, SAH and dyslipidemia, and management of these conditions by pharmaceutical and non-pharmaceutical approaches. Patients who said they did not have these RFs were asked when they were last evaluated for these conditions.

### Sample selection and study population

Based on 2002 analysis^6^ and the 19 cities included in the study, sample selection was performed by cluster sampling, using the census places defined by the Brazilian Institute of Geography and Statistics (IBGE)^8^. The population of each city was divided into three income classes, upper class, middle class and lower class (A, B and C). Whenever possible, results from previous studies involving these cities’ population, generally provided by the prefectures, and data from 2000 IBGE census including data on the mean income per district were used in the study. The study population was drawn from the census places, and then stratified by social class.

In each census place, there was a recruitment list consisting of individuals divided by sex and age. The recruitment was performed by starting from the first house of the census place, and skipping every ten households until the sample was complete. When no respondents were found in the first visit to the household drawn, contact by telephone was first attempted to schedule a visit, and if no answer was obtained, one to three more visits were made at different hours. If no respondent was found, ten other households were skipped.

### Sample calculation and data collection system

Since the study of RFs investigates attributes of a given person (e.g. smoking habit, elevated arterial pressure), the sample size can be calculated by establishing the desired level of confidence, the error percentage (absolute precision), and performing the variability analysis.

Sample size was calculated by PEPI (Programs for EPIdemiologists), version 4.0. By considering an infinite population size, margin of error of 3.5%, a confidence level of 95%, and expected prevalence of 50% (which requires a greater sample size, i.e. considering the worst scenario), the number of subjects required for the study was 784. Since the sample was selected by cluster sampling, the estimated sample size needed to be inflated in order to prevent an increase in the confidence interval. The sample was increased by 50% according to current approaches, which led to a sample of 1,176 individuals.

For a representative sample of Rio Grande do Sul State’s population, its representativeness in the 19 cities was considered for the analysis. For example, the population of Porto Alegre represents approximately 39% of these 19 cities’ population, and hence involved 458 participants (39% of 1,176). The number of individuals recruited in each city is described in Table 1. The final sample size was 1,059 interviewees (statistically representative).

**Table 1:** Cities where data were collected

**Cities**	**Interviewees (n)**
Alegrete	49
Bagé	14
Cachoeira do Sul	30
Caxias do Sul	123
Cruz Alta	16
Erechim	32
Frederico Westphalen	4
Guaíba	30
Ijuí	25
Lajeado	16
Osório	12
Palmeira das Missões	10
Passo Fundo	50
Pelotas	95
Porto Alegre	398
Santa Cruz do Sul	38
Santa Maria	87
Santa Rosa	19
Santo Ângelo	11
Total	1,059

### Data collection and storage

All participants signed an informed consent before entering the study. Whenever necessary, the results of the study and medical counseling will be provided to participants.

Arterial pressure was measured at two moments during the visit, and the second value was recorded. A 5-mL fasting blood sample was collected for lipid and glucose measurements (performed by the ICFUC Clinical Analysis Laboratory). Additionally, body weight, height and abdominal circumference of all participants were measured by the investigators. A Microsoft Access 2007 database was created, and data were digitized and centrally stored at the ICFUC Epidemiology Service.

### Statistical analysis

Qualitative variables are presented as absolute and relative frequency, and quantitative variables as mean and standard deviation. The prevalence of RFs is described with 95% interval confidence. Associations between the prevalence of RFs and the variables “sex” and “year of the study” were analyzed by chi-square test. Analyses were performed using SPSS software version 22, and the level of significance was set at 0.05.

## Results

Twenty-six percent of the study group consisted of older adults (60 years or older), and 57% were women. The prevalence of RFs in 2014 and its comparison with the prevalence in 2002 are described in Table 2. Significant variations were observed in all RFs, notably sedentary lifestyle (which decreased by 1.6-fold). The relative number of current smokers and genetic factors (family predisposition) also decreased from 2002 to 2014. The prevalence of diabetes mellitus (glucose levels higher than 126 mg/dL) in 2014 was not different compared with that in 2002. However, the prevalence of SAH, dyslipidemia and obesity has increased in this period. It is of note that 1.5% of diabetic patients were not under treatment despite being aware of the disease. Similarly, 8.2% of the study group (half of the individuals with dyslipidemia) had dyslipidemia (cholesterol levels greater than 240 mg/dL) and were not receiving treatment despite being aware of the condition. Additionally, 65.8% of the study group had triglyceride levels ≥ 150 mg/dL, 26.8% had HDL levels < 200 mg/dL, and 24.3% LDL > 100 mg/dL. The number of RFs in the same individual was 2, 3 and 4 in 24.6%, 26.3% and 20.6% of participants, respectively. Only 4.9% of the sample (52 individuals) did not have any RF for CAD.

**Table 2:** Comparison in the prevalence of risk factors between 2002 and 2014

**Risk factors**	**2002**		**2014**		**p**
	**%**	CI*	**%**	CI*	
Smoking	33.9	31.0-36.8	23.0	20.5-25.6	< 0.001
Hypertension	31.6	28.8-34.4	39.9	37.0-42.9	< 0.001
Obesity/Overweight	54.7	51.7-57.7	67.7	64.9-70.5	< 0.001
Diabetes mellitus	12.0	5.4-8.6	10.6	8.7-12.4	0.68
Family history	57.3	53.9-60.7	49.7	46.7-52.7	0.001
Sedentary lifestyle	71.3	68.6-74.0	44.2	41.2-47-2	< 0.001
Dyslipidemias†	5.6	4.2-7.0	16.4	14.2-18.7	< 0.001

* CI - Confidence interval;

† total cholesterol ≥ 240 mg/dL.

When the prevalence of RFs was analyzed by gender and by year (Table 3), a difference between genders was observed. In the initial analysis, women had higher cholesterol levels as compared with men (p = 0.04) whereas higher number of current smokers was found among men (p < 0.001). In the new analysis, women were more obese than men (p < 0.001), and men had higher arterial pressure (p < 0.001). The increase of hypercholesterolemic population and decrease of sedentary population in the 12-year interval was not different between genders. However, in general, higher prevalence of obese women (p < 0.001 compared with men) and hypertensive men (p < 0.001 compared with women) was found in 2014. It is worth mentioning that 25% of the sample was composed by individuals aged 60 years or older. Therefore, the increasing prevalence of some RFs is worrying, since it involves the larger proportion of the group composed by younger subjects.

**Table 3:** Comparisons of variables between genders by year and comparisons between years (2002 and 2014) by gender

**Risk factors**	**Year of study**						**Comparison 2002 x2014**	
	**2002**			**2014**			**p**	
	**Women**	**Men**	**p**	**Women**	**Men**	**p**	**Women**	**Men**
Diabetes mellitus	64 (12,3)	59 (12.6)	0.97	52 (8.7)	48 (10.4)	0.39	0.12	0.14
Hypercholesterolemia (200)	151 (29,1)	104 (22.3)	0.04	259 (43.2)	198 (43.0)	0.99	< 0.01	< 0.01
Obesity (BMI > 30)	119 (21,8)	99 (19.6)	0.39	205 (34.2)	108 (23.5)	< 0.01	< 0.01	1.00
Smoking	163 (29,6)	194 (38.0)	<0.01	140 (23.4)	104 (22.6)	0.83	0.02	< 0.01
Sedentary lifestyle	390 (71,3)	361 (71.3)	0.99	278 (46.4)	190 (41.3)	0.11	<0.01	< 0.01
SAH	156 (34,1)	153 (33.3)	0.80	201 (33.6)	222 (48.3)	< 0.01	0.06	< 0.01

Significance level of 5%; p < 0.05 indicates significant differences. BMI: Body mass index; SAH: Systemic arterial hypertension.

## Discussion

CVDs represent a major threat to health, and the control of RFs seems to be the most logical way to prevent them. It is quite evident that the RFs in this study are responsible for the development of CVD^19,20^. Our study demonstrated that the prevalence of RFs remains increased in Rio Grande do Sul State. Although a decrease in the prevalence of sedentary lifestyle and smoking has occurred, SAH, dyslipidemia and obesity are still worrying, differ between men and women, and are independent of socio-economic classes. The seven RFs analyzed will be discussed below.

### Sex and age

The influence of RFs for CVDs is different in men and women^21,22^. Our study demonstrated that in Rio Grande do Sul State, obesity was more prevalent among women and hypertension among men. Obesity and hypertension are closely related to the development of arteriosclerosis^12,21^. Taking into account sex and age, plaque rupture is more likely for older women^21^. Also, visceral fat accumulation is more common in women than in men, due to hormonal conditions or life habits.

Consumption of barbecue is embedded in the lifestyle of men in Rio Grande do Sul State, contributing to excessive salt and fat intake, and possibly relating to the increased prevalence of SAH in this population. Due to their importance, obesity and hypertension will be discussed separately.

### Smoking

Two recent studies indicate an increased risk for CVD in active and passive smokers^23,24^. A multiethnic study^25^ revealed that predictive factors of atherosclerotic diseases, such as increased levels of high-sensitive C-reactive protein, are found in smokers free of CVD. In addition, the authors demonstrated that smokers have a 70% higher chance of developing coronary atherosclerosis as compared with nonsmokers^25^. Individuals who smoke two or three packs of cigarettes a day have a two-fold to three-fold greater risk. Smoking increases platelet adhesion, causes arterial endothelial injury and contributes to elevated blood pressure (by inducing arterial thickening and promoting smooth cell proliferation), additionally to increase total cholesterol and LDL levels, and decrease HDL levels^26^. Interestingly, our study demonstrated that after 12 years, the number of active smokers has decreased in Rio Grande do Sul State. Anti-smoking campaigns and the Brazilian legislation prohibiting smoking in workplaces and enclosed public places may have contributed to the decrease of this RF.

### Hypertension

SAH is considered the main RF for the development of CVDs. Determination of cardiovascular risk depends on the stage of hypertension, presence of cardiovascular RFs and target organ damage, and associated clinical conditions^17,18,26^. SAH is also an important cause of systolic heart failure in developing countries including Brazil^26^. SAH contributes to atherogenic plaque formation, increasing the risk of cardiovascular events by two to three times^27^. Non-refractory hypertension is controlled by antihypertensive drugs, which has positively affected cardiovascular morbidity and mortality^27^.

The present study demonstrated that the prevalence of SAH in Rio Grande do Sul State in 2014 is higher than that in 2002. Such increase may be due, in part, to the increased prevalence of obesity, overweight, and dyslipidemia, since these factors contribute to elevation in blood pressure. Therefore, new campaigns about blood pressure control and the influence of some factors such as high-sodium diet on blood pressure are required for the population in the south of Brazil. In fact, low education affects the prevalence of SAH and awareness about this condition, confirming the existence of a relationship between social inequality and health inequality^10^.

### Obesity

Obesity is a comorbidity that induces chronic inflammatory states, and lead to a predisposition of other RFs, including dyslipidemia, SAH and diabetes, which in turn, further increase the risk of cardiovascular problems^28^. Obesity is considered a global epidemic that is related to the development of metabolic syndrome^9^. According to Rio Grande do Sul State’s Secretary of Health, two million people have metabolic syndrome and other obesity-related disorders, which increase the risk of myocardial infarction or stroke by three times^29^.

The causes of obesity vary from inadequate food intake and low physical activity to psychological problems such as depression and anxiety, leading to increased risk for other diseases including diabetes and heart diseases. Our study demonstrated that the prevalence of obesity increased from 2002 to 2014, and that women are more commonly affected than men.

### Diabetes mellitus

Diabetes mellitus has become an important RF in face of progressive body weight gain, growth of the elderly population, inadequate food intake and longer life expectancy. Mortality rates in diabetes are higher than in general population. Acute ischemia, when associated with diabetes, accounts for 80% of all causes of mortality in these patients, and approximately 75% of hospitalizations for diabetes complications^30^. Diabetes is particularly harmful to women, especially in the presence of low HDL levels, which confer higher risk for CAD as compared with men in the same condition^31^.

In the Brazilian Longitudinal Study of Adult Health (ELSA-Brasil)^32^, the prevalence of diabetes was 19.7%, 50.4% of them previously undiagnosed. The incidence was higher in the elderly, in obese subjects, non-whites, and those with less formal education. Ramos et al^33^ concluded that fasting plasma glucose was an independent predictor of major cardiac events, including death, reinfarction and revascularization in elderly patients at early stage of acute coronary syndrome.

Our findings show that the prevalence of diabetes mellitus is relatively low (nearly 10%) in Rio Grande do Sul State, and despite not statistically significant, the prevalence was lower in 2014 compared with 2002. However, due to its close relationship with CAD and other RFs, the investigation of diabetes mellitus should be included in future studies on the management of CVD.

### Family history

Family history of sudden death or premature heart attack, diabetes, SAH and dyslipidemia, particularly in first-degree relatives, indicate an increased risk of coronary disease^27^. In addition to genetic predisposition, family background may be related to unhealthy lifestyle^27,34^. Although still high (approximately 50%), our study demonstrated that the prevalence of this RF in 2014 was lower than in 2002. Nowadays, family history may be related to healthier life habits, including physical exercise (since sedentary lifestyle significantly decreased in the period), which lead to a better quality of life and provide an unfavorable environmental condition for the expression of disease-related genes.

### Sedentary lifestyle

Physical activity is strongly recommended and encouraged for secondary prevention of CVD^34^, since sedentary behavior may be a greater risk factor for coronary heart disease than the combination of high cholesterol level, hypertension and smoking; the higher the level of physical activity, the lower the risk of the disease^34^. Physical inactivity increases the risk of obesity, hypertension, and type II diabetes, causes a decrease in HDL levels, and has a direct effect on atheroma plaque growth^35^. An interesting finding of this study was the significant decrease in the prevalence of sedentary lifestyle in the study population, which may be caused by several factors including greater access to information and scientific advances on the positive effects of exercise on human health.

### Dyslipidemias

Elevated cholesterol is one of the major RFs for CVDs, and statins, used to lower cholesterol levels, are closely associated with reduction of atherosclerosis^36^. In our study, both hypercholesterolemia (total cholesterol > 200 mg/dL) and dyslipidemia (total cholesterol > 240 mg/dL) were highly prevalent in the study group, and increased from 2002 to 2014. Unfortunately, part of the group with high cholesterol levels was not being treated despite being aware of the condition. Therefore, informative campaigns and adoption of secondary prevention measures are necessary in Rio Grande do Sul State. Among the strategies, physical activity may contribute to decrease systemic levels of cholesterol^35^. Fernandes et al^37^ reported that the prevalence of dyslipidemia has increased in several parts of Brazil, however, the level of physical activity required for a beneficial effect on plasma lipoprotein levels is still uncertain.

In developing countries, six RFs: SAH, smoking, high glucose levels, sedentary lifestyle, overweight/obesity and dyslipidemias contribute substantially to death from CVDs^12^. The INTERHEART study indicated that RFs account for more than 90% of all causes of CVDs^12^, and contribute to death from metabolic disorders and cancer^38^. Except for diabetes and hypercholesterolemia, all other RFs are significantly more prevalent in socio-economically vulnerable populations^13^. Previous studies conducted in Rio Grande do Sul State, on populations from the cities of Porto Alegre^3,10,28^, Passo Fundo^39^ and Pelotas^11,12^, have drawn attention to the high prevalence and association between RFs in the elderly population, suggesting the need for immediate diagnosis and management of the risks.

The study has some limitations, including the fact that the sample size was reduced from 1,176 (initially calculated) to 1,059, and some of the interviewers and data collectors needed to be replaced, causing a delay in the acquisition of data. However, since the objective and methodology of the current study were the same as those of the study conducted in 2002^6^, one can assume that comparisons in the RFs prevalence between both studies yield their evolution in a 12-year period. This is the first study that allows a comparative analysis of the prevalence of RFs in a state in the south of Brazil. The study showed a change, either an increase or a decrease, in the prevalence of all RFs in the study populations. Although the Rio Grande do Sul State’s population has adopted healthier habits, such as physical activity and smoking reduction, they still need to receive information, on an ongoing basis, about other RFs such as SAH, dyslipidemia, diabetes and obesity. These factors have complex etiology and pathophysiology, requiring various preventive measures. However, the study also showed that many individuals with RFs for CVDs, despite being aware of their condition, do not seek treatment or abandon it, and many of these preventive actions become partially effective. It is evident that such complex diseases with still unknown mechanisms require nonpharmaceutical and pharmaceutical, preventive and curative therapies. RF control is expensive, requires patients’ perseverance, medical follow-up, and awareness programs for the population.

## Conclusion

1. The prevalence of RFs for CADs remains high in Rio Grande do Sul State after 12 years.
2. Differences in the RFs prevalence between both studies (2002 and 2014) indicate the trend of these factors and the necessity of preventive programs, and more effective and efficient strategies.
3. Family history, sedentary lifestyle and smoking decreased, whereas dyslipidemia, hypertension and obesity increased in the study population.
4. The prevalence of obesity was higher in women, while the prevalence of hypertension was higher in men.
5. Future studies to further and better evaluate the importance of socioeconomic status, school attainment, and diet quality as RFs for CVDs are needed.
